# The α_2_ Na^+^/K^+^-ATPase isoform mediates LPS-induced neuroinflammation

**DOI:** 10.1038/s41598-020-71027-5

**Published:** 2020-08-25

**Authors:** J. A. Leite, T. J. Isaksen, A. Heuck, C. Scavone, K. Lykke-Hartmann

**Affiliations:** 1 Department of Biomedicine, Aarhus University, Aarhus, Denmark; 2 Department of Pharmacology, Instituto de Ciências Biomédicas, Universidade de São Paulo, São Paulo, Brazil; 3 Department of Pharmacology, Instituto de Ciências Biológicas, Universidade Federal de Goiás, Goiânia, Brazil; 4 Department of Clinical Medicine, Aarhus University, 8000 Aarhus C, Denmark; 5 Department of Clinical Genetics, Aarhus University Hospital, 8200 Aarhus N, Denmark

**Keywords:** Gene regulation in immune cells, Neuroimmunology, Neurological disorders

## Abstract

Na^+^/K^+^-ATPase is a transmembrane ion pump that is essential for the maintenance of ion gradients and regulation of multiple cellular functions. Na^+^/K^+^-ATPase has been associated with nuclear factor kappa B (NFκB) signalling, a signal associated with lipopolysaccharides (LPSs)-induced immune response in connection with activated Toll-like receptor 4 (TLR4) signalling. However, the contribution of Na^+^/K^+^-ATPase to regulating inflammatory responses remains elusive. We report that mice haploinsufficient for the astrocyte-enriched α_2_Na^+^/K^+^-ATPase isoform (α_2_^+/G301R^ mice) have a reduced proinflammatory response to LPS, accompanied by a reduced hypothermic reaction compared to wild type litter mates. Following intraperitoneal injection of LPS, gene expressions of *Tnf-α*, *Il-1β*, and *Il-6* was reduced in the hypothalamus and hippocampus from α_2_^+/G301R^ mice compared to α_2_^+/+^ littermates. The α_2_^+/G301R^ mice experienced increased expression of the gene encoding an antioxidant enzyme, NRF2, in hippocampal astrocytes. Our findings indicate that α_2_Na^+^/K^+^-ATPase haploinsufficiency negatively modulates LPS-induced immune responses, highlighting a rational pharmacological target for reducing LPS-induced inflammation.

## Introduction

The Na^+^/K^+^-ATPase is an integral membrane protein that spans the entirety plasma membrane of all animal cells. It exchanges three Na^+^ ions out of the cell for two K^+^ ions into the cell using energy generated from ATP hydrolysis^1^. The Na^+^/K^+^-ATPase activity is important for many cellular functions, such as maintenance of membrane potentials, cellular volume regulation and pH adjustments, and supporting secondary transport of transmitters^2^.

Studies have shown differences in the physiological functions of NKA isoforms. The α1-Na^+^/K^+^-ATPase isoform also appears to act as a receptor, a signal transducer, and a cell adhesion molecule^3^, involving various pathway components such as the membrane-associated non-receptor tyrosine kinase Src pathway^4^, Ras/Raf/ERK1/2 pathway activation^5^, the phosphate inositol 3-kinase (PI_3_K) pathway, the PI_3_K-dependent protein kinase B pathway, phospholipase C, [Ca^2+^]_i_ oscillations^6–8^, and gene transcription^9,10^. Interestingly, Na^+^/K^+^-ATPase-mediated increases in Ca^2+^ concentration can affect gene transcription by promoting the translocation of nuclear factor-kappa B (NFκB) from the cytosol to the nucleus and by phosphorylating cAMP response element binding protein (CREB)^11^. However, elegant studies have shown that the a_2_ Na^+^/K^+^-ATPase isoform does not interfere with the signaling and activation of the Src, ERK and PI3K/Akt pathways^12,13^. In addition, it is important to highlight the isoforms of Na^+^/K^+^-ATPase are located in distinct regions in the plasma membrane of cells^14^, which confer different physiological functions in the regulation of intracellular Na^+^ and Ca^2+^. Within this context, studies with different cell types^15^, such as aortic smooth muscle and astrocytes, have demonstrated the importance of the a_2_Na^+^/K^+^-ATPase isoform in the maintenance of intracellular Na^+^, as well as the interaction of the α_2_ isoform with the Na^+^/Ca^2+^ exchanger for the reaction of intracellular Ca^2+^ levels and their signaling^16,17^. Furthermore, ifenprodil, an *N*-methyl-d-aspartate receptor antagonist, has been shown to restore GDNF‐evoked Ca^2+^ transients that are attenuated by LPS by inducing Na^+^/K^+^-ATPase expression^18^.

In mammals, four different Na^+^/K^+^-ATPase α isoforms (α_1−4_) are expressed, of which α_1−3_ are found in the central nervous system. While α_1_ is ubiquitously expressed and thought-about to maintain housekeeping cellular functions, the α_2_ isoform is functional primarily in astrocytes and developing neurons and the α_3_ isoform is restricted to neurons^19,20^.

In the adult brain, the Na^+^/K^+^-ATPase α_2_ isoform is enriched in astrocytes and important for extracellular K^+^ clearance and function to support glutamate uptake from the synaptic cleft^21–23^ and impair astrocytic K^+^ clearance^24^. Accordingly, mutations in the *ATP1A2* gene, which encodes the α_2_ subunit isoform of the Na^+^/K^+^-ATPase, can cause familial hemiplegic migraine type 2 (FHM2)^25^, a subtype of migraine with aura^26^. Several *Atp1a2* gene-modified mouse models have been made and extensively used to study the α_2_ isoform in vivo^25^. While KO and KI homozygous mice dies immediately after birth^27–30^ heterozygous α_2_ knock-out (KO) and knock-in (KI) mice are viable.

FHM2 patients with the G301R mutation resulting from a gene variant in the *ATP1A2* gene have migraine comorbidity with epilepsy, coma, motor symptoms and psychiatric disorders such as depression and obsessive–compulsive disorder (OCD)^31,32^. Heterozygous KI mice containing the G301R disease mutation (α_2_^+/G301R^ mice) displayed FHM2-related phenotypes, including mood depression and obsessive–compulsive disorder (OCD)-like symptoms^33^, besides showed a greater susceptibility to epilepsy and disseminated cortical depression (CSD)^33,34^. Moreover, astrocyte-neuron primary in vitro cultures from α_2_^(G301R/G301R)^ mice revealed impaired glutamate uptake^33^. Surprisingly, when submitted to spinal cord injury, the α_2_^+/G301R^ mice display improved functional recovery and decreased lesion volume compared to littermate controls (α_2_^+/+^)^35^. These phenotypes were associated with changes in pro- and anti-inflammatory cytokines, with the cytokines TNF, IL-6, and IL-10 upregulated in the spinal cord of α_2_^+/G301R^ and α_2_^+/+^ mice with a spinal cord injury^35^. Interestingly, the functional recovery of the α_2_^+/G301R^ mice was improved compared to α_2_^+/+^ mice and correlated with a significantly reduced lesion size^35^. In line with this, astrocytes deficient of the mutant superoxide dismutase 1 (SOD1) that also was depleted from the α_2_Na^+^/K^+^-ATPase, were able to protect motor neurons from degeneration in co-cultured primary motor neurons^36^. In these SOD-deficient astrocytes, mitochondrial respiration and inflammatory gene expressions appeared induced.

The genetic mechanisms of the *Atp1a2* pathophysiology thus appear to reply on the ion balance, however, the impact of this on the immune response remains unknown.

As an initial attempt to understand this, we challenged heterozygous α_2_^+/G301R^ mice by lipopolysaccharides (LPS) administration. We hypothesised that the mice would be compromised compared to wild type litter mates as ion channels-related diseases contribute to similar symptoms, and can be used as potential targets to modulate immune response and to treat inflammatory disorders and cancer.

LPSs, known as lipoglycans and endotoxins, acts as a prototypical endotoxin, binding the CD14/TLR4/MD2 receptor complex in many cell types, which promotes the production and secretion of proinflammatory cytokines, nitric oxide, and eicosanoids. LPS can induce neuroinflammation in the brain, including NF-κB activation in rodents, which can lead to impaired cognitive performance^37,38^.

Interestingly, short interference (si)RNA-mediated knockdown of the gene encoding the α_2_ isoform in primary astrocyte cultures prevented the LPS-mediated activation of ERK and NFκB^39^, but the precise mechanism through which the α_2_Na^+^/K^+^-ATPase pump regulates neuroinflammation remains elusive.

Here, we studied the α_2_Na^+^/K^+^-ATPase regulation of LPS-mediated neuroinflammation in the hypothalamus and hippocampus using a heterozygous mouse model (α_2_^+/G301R^) that exhibits α_2_ haploinsufficiency^33^. We found a reduction in the systemic production of the proinflammatory cytokines TNF-α, IL-1β, and IL-6 following LPS administration, as well as a reduced hypothermic response in α_2_^+/G301R^ mice.

## Results

### α_2_Na^+^/K^+^-ATPase haploinsufficiency reduce liposaccharide (LPS)-induced inflammation in vivo

**Figure 1:**
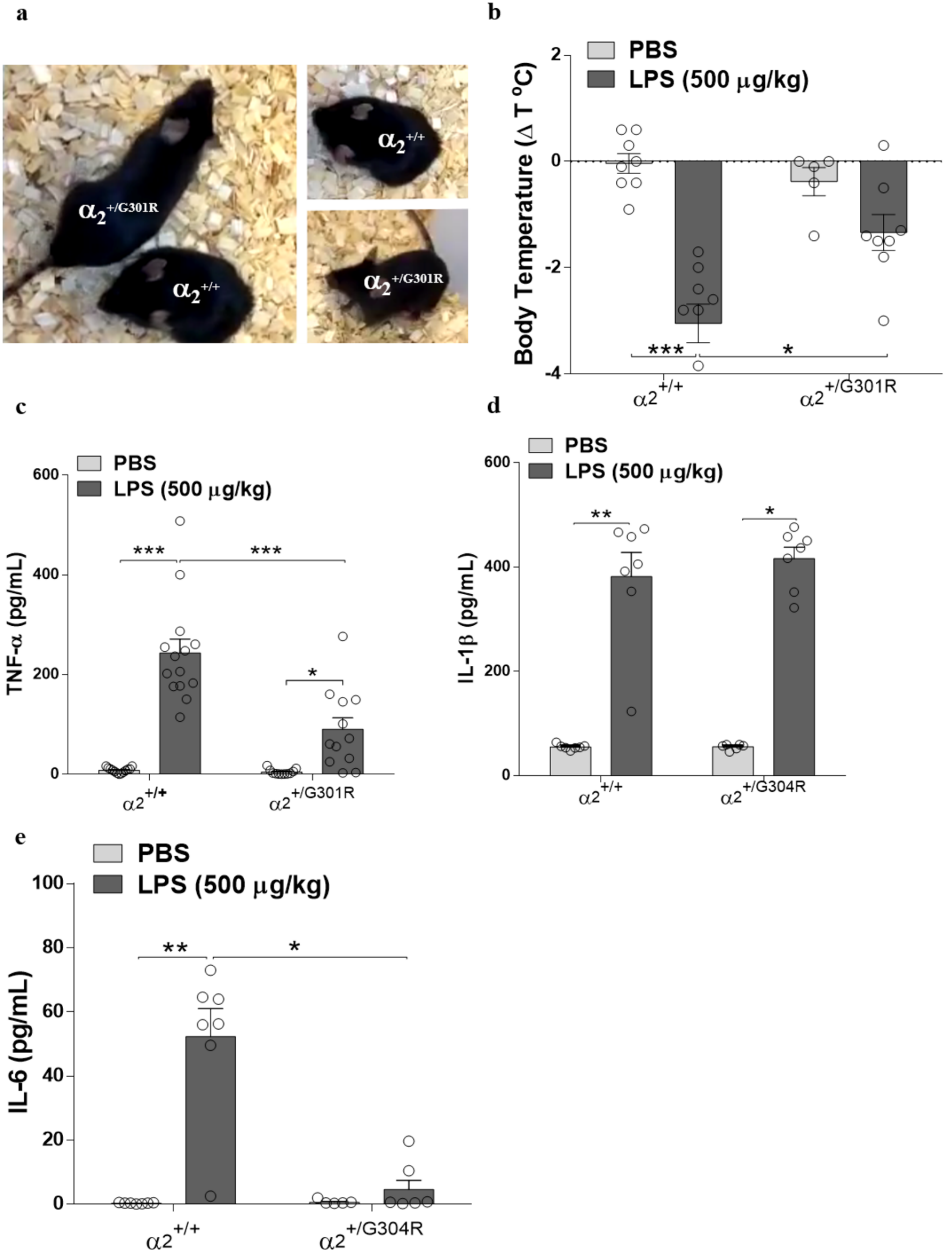
α_2_^+/G301R^ mice display decreased sickness behaviour. α_2_^+/G301R^ mice experience hypothermia, and systemic proinflammatory cytokine levels compared to α_2_^+/+^ mice following LPS administration (500 µg/kg, intraperitoneally (IP) injected). (**a**) Example of sickness behaviour 4 h after LPS administration. As illustrated, the α_2_^+/+^ mice displayed sickness behaviour, less locomotion, and, briefly, hypothermia, in contrast to the α_2_^+/G301R^ mice that displayed less sickness behaviour after LPS administration. (**b**) The differences in body temperature 4 h after LPS administration compared to baseline (t = 0) in the α_2_^+/+^ and α_2_^+/G301R^ animals treated with PBS or LPS, with a significant different after LPS treatment. (**c**–**e**) The α_2_^+/G301R^ mice and their WT littermates (α_2_^+/+^) were injected with saline or LPS (500 µg/kg), and the levels of TNF-α (**c**) (*n* = 12 for the α_2_^+/+^ mice and *n* = 14 for α_2_^+/G301R^ mice), IL-1β (**d**) [α_2_^+/+^ (*n* = 6) and α_2_^+/G301R^ mice (*n* = 7)] and IL-6 (**e**) [α_2_^+/+^ (*n* = 6) and α_2_^+/G301R^ (*n* = 7)] were measured in the serum 4 h after saline/LPS treatment. The data are presented as the mean ± SEM. *p < 0.05, **p < 0.01, ***p < 0.001 (Kruskal–Wallis test followed by Dunn’s post hoc test).

To assess the effect of LPS in mice that are haploinsufficient for the α_2_ isoform, we used a knockin mouse model containing the FHM2-associated G301R disease mutation, which has been shown to harbour haploinsufficiency in heterozygotes α_2_^+/G301R^ mice, associated with FHM2 traits^33^ . At such, α_2_^+/G301R^ and α_2_^+/+^ mice were intraperitoneally injected with LPS (500 µg/kg). Intriguing, the LPS-injected α_2_^+/G301R^ mice appeared much less affected by LPS and exhibited normal cage roaming in contrast to the α_2_^+/+^ littermates, which exhibited the expected severe sickness behaviour after LPS treatment (Supplementary Data, movie 1) (Fig. 1a).

To test if the apparently unaffected LPS-treated α_2_^+/G301R^ mice experienced changes in body temperature, a characteristic effect of LPS treatment, body temperatures were measured at baseline and 4 h after LPS injection. In line with the observed phenotypes, the α_2_^+/+^ mice exhibited a severe drop in body temperature (− 3.85 ± 3 °C) after LPS administration, whereas the α_2_^+/G301R^ mice exhibited a significantly smaller drop in body temperature (− 1.40 ± 3.3 °C) (Fig. 1b). To evaluate whether cytokine levels were altered in the mice treated with LPS and if these alterations were different in the α_2_^+/G301R^ mice, blood samples were collected 4 h after LPS administration and cytokine levels were measured using an enzyme-linked immunosorbent assay (ELISA). As expected, the LPS-treated α_2_^+/+^ mice exhibited significantly increased serum tumour necrosis factor-α (TNF-α), interleukin-1 beta (IL-1β) and IL-6 levels compared with those exhibited by the PBS-treated α_2_^+/+^ mice (Fig. 1c–e). The LPS-treated α_2_^+/G301R^ mice had significantly reduced serum levels of TNF-α (Fig. 1c), and IL-6 (Fig. 1e) compared with those of the LPS-treated α_2_^+/+^ mice. The levels of IL-1β in the LPS-treated α_2_^+/G301R^ mice were equal to those in the LPS-treated α_2_^+/+^ mice (Fig. 1d).

Combined, these results show a reduced sickness behavior and hypothermic response observed in the α_2_^+/G301R^ mice compared to α_2_^+/+^ mice, and moreover, indicate that induction of TNF-α and IL-6 is compromised in response to LPS treatment in α_2_^+/G301R^ mice. Our data show that LPS challenge promoted a serum increase of IL-1β that was not reversed in α_2_^+/G301R^ mice, thus suggesting that α_2_ activity does not interfere with the release of IL-1β in its mature form.

### α_2_Na^+^/K^+^-ATPase is not required for LPS-induced cytokine production in macrophages

Macrophages are the main producers of proinflammatory cytokines, including IL-6 and TNF-α^40,41^. Therefore, next we studied whether the decreased systemic levels of these cytokines in α_2_^+/G301R^ mice were due to a reduced macrophage response and, hence, whether the macrophage response to LPS involves the Na^+^/K^+^-ATPase α_2_ isoform. To this end, we measured TNF-α production in response to LPS treatment in bone marrow-derived macrophages (BMDMs) isolated from α_2_^+/G301R^ mice and wild-type (WT) littermates.

**Figure 2:**
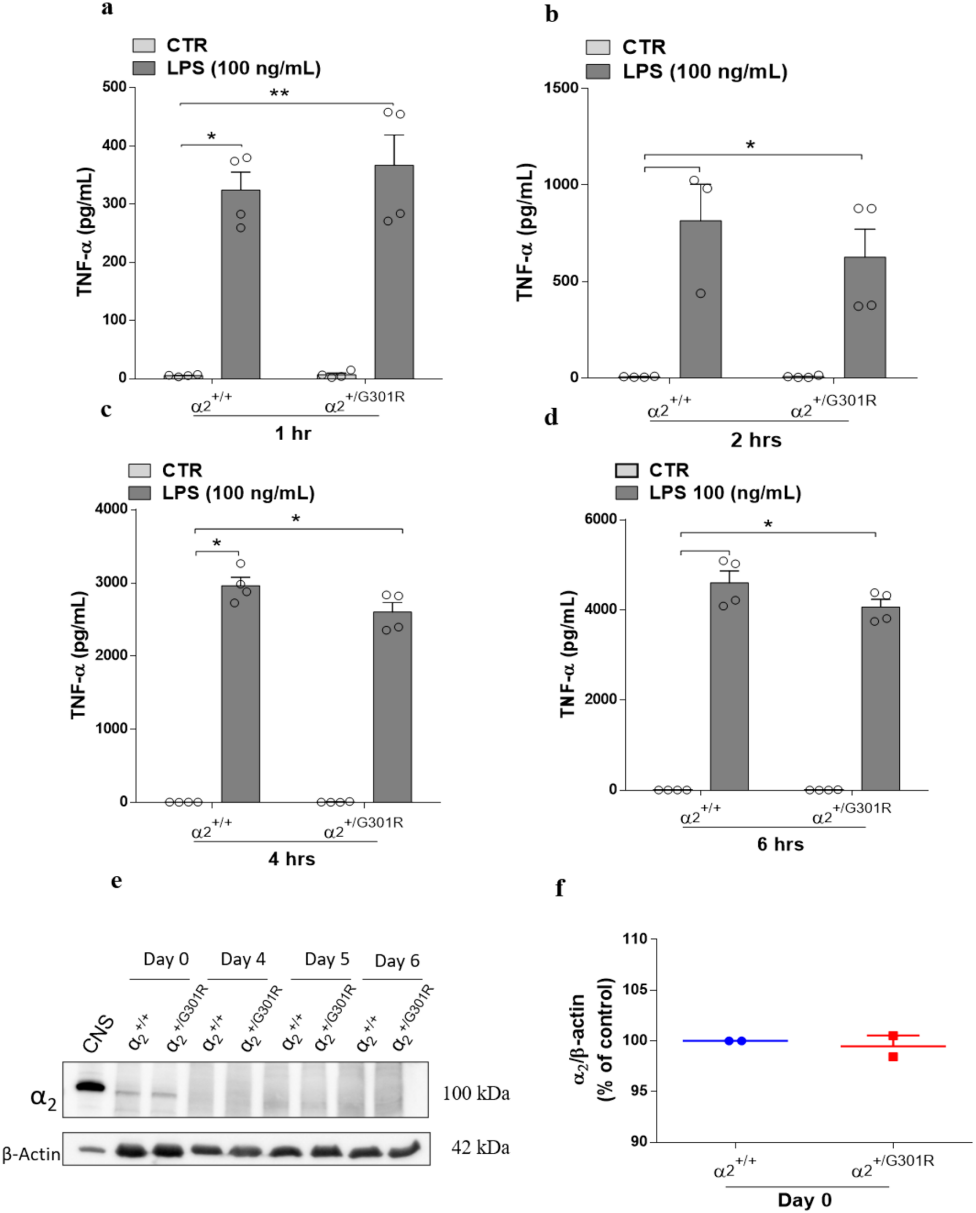
The α_2_ Na^+^/K^+^-ATPase isoform is not involved macrophage cytokine production in response to LPS. BMDMs derived from bone marrow cells from the femurs and tibias of α_2_^+/+^ and α_2_^+/G301R^ mice were cultured in L929 cell supernatant for 6 days. After 6 days, the BMDMs were treated with LPS (100 ng/mL) or PBS (CTR). (**a**–**d**) The levels of TNF-α were measured in the supernatant 1 h (**a**), 2 h (**b**), 4 h (**c**), and 6 h (**d**) after LPS treatment. (**e**) α_2_ Isoform levels were measured by Western blotting in bone marrow derived macrophages from α_2_^+/+^ and α_2_^+/G301R^ mice at the indicated times during differentiation. β-Actin was used as a loading control. A protein extract from the central nervous system (CNS) was loaded as a positive control. (**f**) Densitometric analysis from day 0 (arbitrary units, A.U.) showing that no obvious differences existed in the α_2_ protein levels in the BMDMs derived from the α_2_^+/+^ and α_2_^+/G301R^ mice. The data are presented as the mean ± SEM from two individual experiments. The data are presented as the mean ± SEM. *p < 0.05 and **p < 0.01 (Kruskal–Wallis test followed by Dunn’s post hoc test).

Surprisingly, the levels of TNF-α in the BMDMs from both α_2_^+/+^ and α_2_^+/G301R^ did not differ significantly at any of the time points analysed (Fig. 2a–d). However, it is noteworthy that although the levels of TNF-α did not differ between α_2_^+/+^ and α_2_^+/G301R^, in both cases they are significantly increased after 1, 2, 4 and 6 h of LPS treatment. This suggested that the Na^+^/K^+^-ATPase α_2_ isoform may not be functionally required for LPS-induced TNF-α release in macrophages. Indeed, low levels of α_2_ protein were detected by Western blotting in BMDM cells and the levels diminished during BMDM differentiation in vitro (Fig. 2e and Supplementary Fig. 1). Furthermore, no significant differences in α_2_ isoform expression in the α_2_^+/+^ and α_2_^+/G301R^ BMDM cells were observed (Fig. 2f), suggesting a low basal level of α_2_ protein not significantly affected by the α_2_ haploinsufficiency. Under these conditions, this implies that the α_2_Na^+^/K^+^-ATPase might regulate the proinflammatory response to LPS in other cell types.

### LPS-induced neuroinflammation is suppressed in the hippocampus and hypothalamus of heterozygous α_2_^+/G301R^ mice

Because steroid ouabain (OUA), a Na^+^/K^+^-ATPases inhibitor, has an anti-inflammatory effect in the rat hippocampus^42–44^, and because the hypothalamus endocrinologically regulates temperature control, we examined the α_2_ protein level in the hippocampus and hypothalamus of α_2_^+/G301R^ mice.

**Figure 3:**
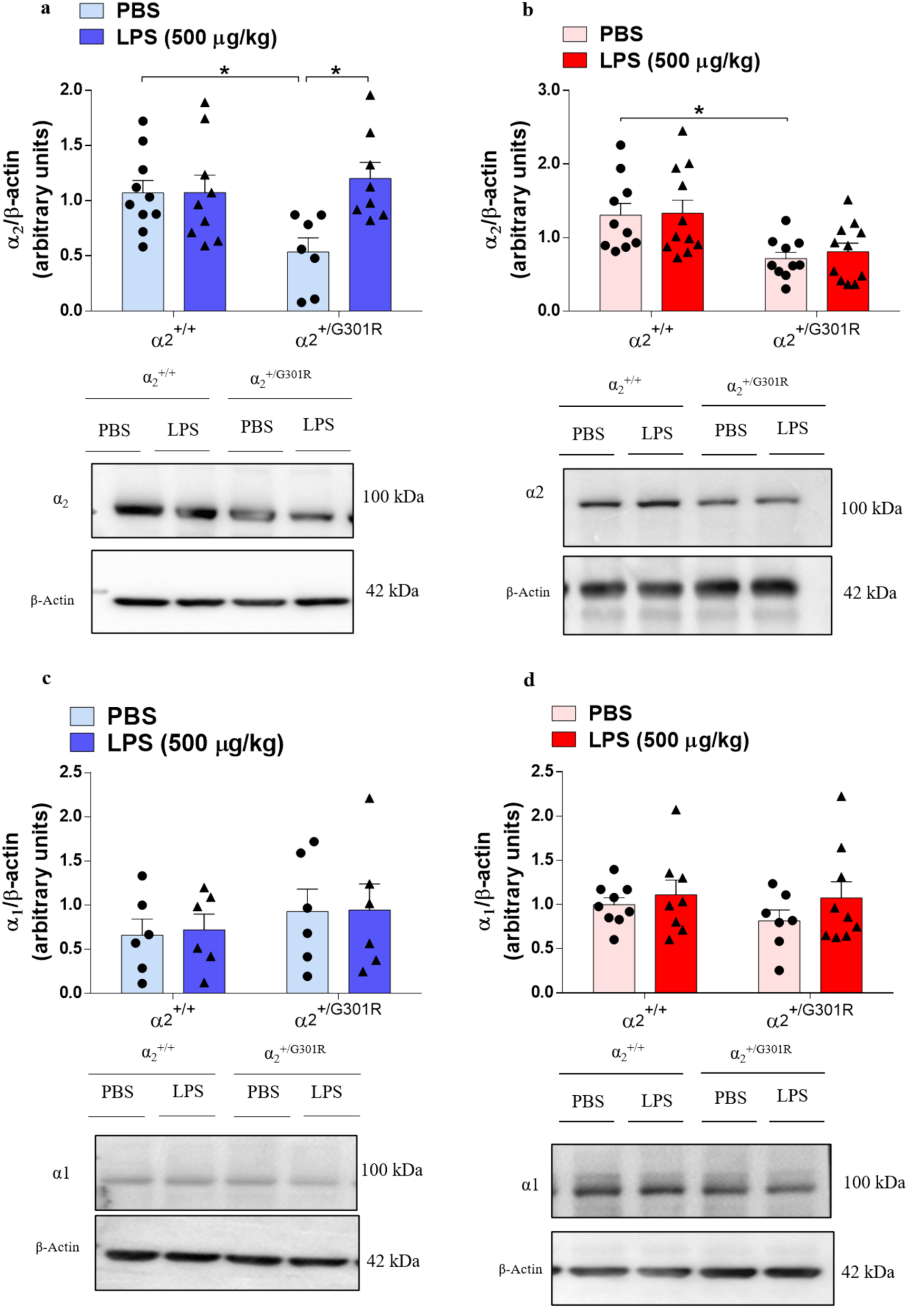
The levels of α_2_Na^+^/K^+^-ATPase isoform are reduced in the hippocampus of α_2_^+/G301R^ mice. α_1_ and α_2_ isoform protein levels assessed by Western blotting in the hypothalamus (blue bars) and hippocampus (red bars). (**a**,**c**) Representative digital images of Western blots and densitometric analysis (arbitrary units) of hypothalamic lysates from the α_2_^+/+^ and α_2_^+/G301R^ mice 4 h after saline or LPS injection showed no differences in α_1_Na^+^/K^+^-ATPase (**a**) (*n* = 8–10 mice/group) and α_2_Na^+^/K^+^-ATPase (**c**) (*n* = 6 mice/group). (**b**,**d**) Representative digital images of Western blots and densitometric analysis of hippocampal lysates from the α_2_^+/+^ and α_2_^+/G301R^ mice 4 h after saline or LPS injection showed no differences in α_2_Na^+^/K^+^-ATPase (**b**) (*n* = 10–11 mice/group) but showed a reduction in α_2_Na^+^/K^+^-ATPase irrespective of LPS treatment (**d**) (*n* = 7–9 mice/group). The results are expressed relative to the control (PBS) as the mean ± SEM from three individual experiments. *p < 0.05 (Kruskal–Wallis test followed by Dunn’s post hoc test).

No significant changes were observed in the levels of the α_2_ isoform in the hypothalamus between the α_2_^+/G301R^ and α_2_^+/+^ mice (Fig. 3a, Supplementary Fig. 2). Like the observations in macrophages, this may imply that the α_2_ isoform may not be produced at levels that would permit a detectable difference. By contrast, the α_2_ isoform levels were significantly reduced in the hippocampus in the α_2_^+/G301R^ mice compared with the α_2_^+/+^ mice (Fig. 3b, Supplementary Fig. 3). As a control, we also examined the levels of the housekeeping α_1_ isoform. No differences were detected in the α_1_ isoform levels in the hypothalamus and hippocampus (Fig. 3c,d, Supplementary Fig. 4, 5), confirming that the housekeeping α_1_ isoform is not affected by the G301R mutation in the α_2_ isoform. Moreover, LPS injection did not influence the levels of the α_1_ (Fig. 3c,d) or α_2_ (Fig. 3a,b) proteins in the hypothalamus or hippocampus in either the α_2_^+/+^ or α_2_^+/G301R^ animals compared to those in the PBS-treated littermates.

Since a significant reduction of α_2_ proteins levels was observed in hippocampus, but no difference in the hypothalamus, we next determined it this would influence the gene expression of *TNF-α*, *IL-1β,* and *IL-6* genes.

**Figure 4:**
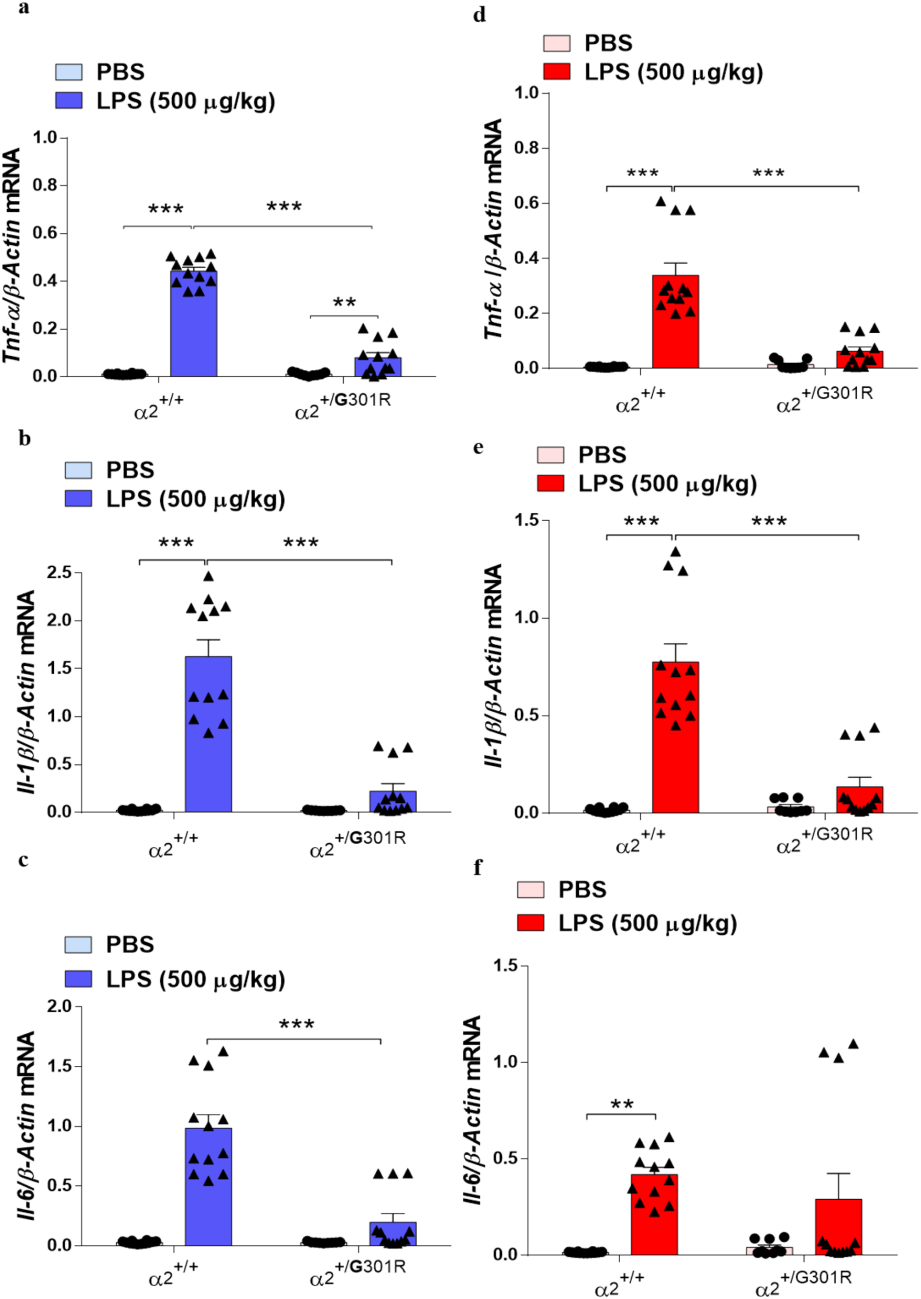
The levels of cytokines are reduced in the hippocampus and hypothalamus of α_2_^+/G301R^ mice. α_2_Na^+^/K^+^-ATPase haploinsufficiency decreases lipopolysaccharide (LPS)-induced hypothalamic (blue bars) and hippocampal (red bars) cytokine transcription. (**a**–**f**) TaqMan quantitative PCR analysis of *TNF-α* (**a**,**d**); *IL-1β* (**b**,**e**); and *IL-6* (**c**,**f**) gene expression relative to the expression of β*-*actin, used as endogenous reference gene. LPS induced significant increases in the levels of TNF-α in the hypothalamus of both α_2_^+/+^ (*n* = 3), and α_2_^+/G301R^ (*n* = 4), mice. LPS induced significant increases in the levels IL-1β and IL-6 in the hypothalamus (blue bars) of α_2_^+/+^ mice (*n* = 3), but not in α_2_^+/G301R^ (*n* = 3) mice. In the hippocampus (red bars), LPS induced significant increases in the levels of TNF-α, IL-1β and IL-6 of α_2_^+/+^ mice (*n* = 3), but not in α_2_^+/G301R^ (*n* = 4) mice. All data are presented as the mean ± SEM. *p < 0.05, **p < 0.01, ***p < 0.001 (two-way ANOVA followed by Tukey’s post hoc test*.*

Cytokine expression was measured specifically in the hypothalamus and hippocampus of the α_2_^+/+^ and α_2_^+/G301R^ mice after PBS or LPS treatment by reverse transcription quantitative polymerase chain reaction (RT-qPCR). As expected, LPS treatment significantly upregulated the transcription of *Tnf-α*, *Il-1β,* and *Il-6* genes in the hypothalamus (Fig. 4a–c) and hippocampus (Fig. 4d–f) of the α_2_^+/+^ mice compared with the PBS control group. By contrast, the transcription of these genes in the hypothalamus and hippocampus of the α_2_^+/G301R^ mice was unaffected by LPS treatment compared with that in the PBS-treated littermates (Fig. 4a–f).

This indicates that part of the lack of a LPS immune response is associated with a significant reduced expression of the *TNF-α*, *IL-1β,* and *IL-6* genes in both hippocampus and hypothalamus.

### *Tlr4* mRNA expression is reduced in astrocytes from α_2_^+/G301R^ mice treated with LPS

**Figure 5:**
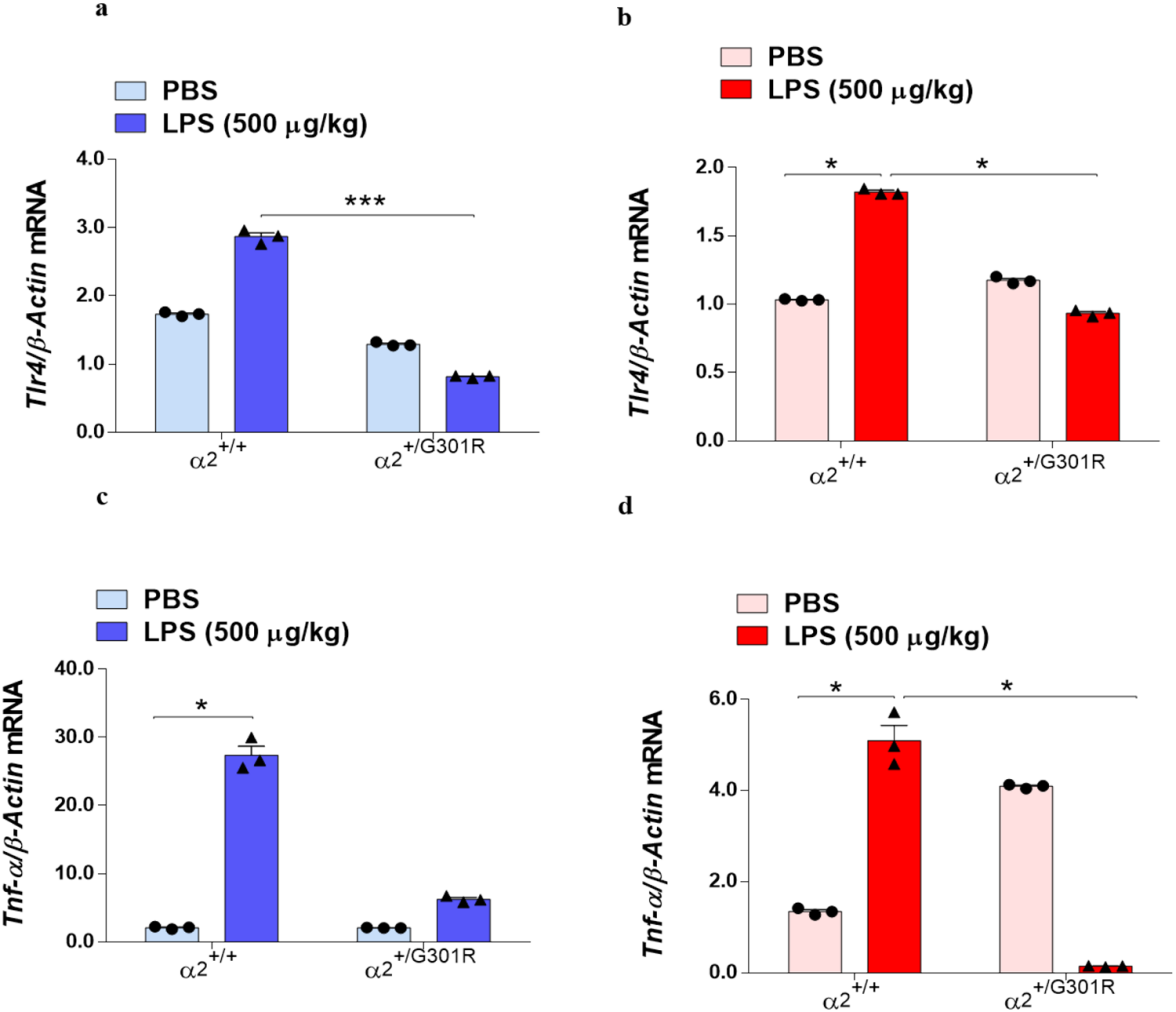
The level of *Tlr4* is reduced in the hippocampus and hypothalamus of α_2_^+/G301R^ mice. The α_2_^+/G301R^ mice exhibited reduced *Tlr4* and *Tnf-α* mRNA expression in astrocytes isolated from the hypothalamus (blue bars) and hippocampus (red bars) compared with that in the α_2_^+/+^ mice after PBS treatment. (**a**,**b**) qPCR analysis of *Tlr4* relative to β-actin expression in the hypothalamus (**a**) and hippocampus (**b**) showed a significant reduction of *Trl4* in these brain areas, notably after LPS treatment, between the α_2_^+/+^ (*n* = 3) and α_2_^+/G301R^ (*n* = 3) animals. The qPCR analysis of *Tnf-α* relative to β-actin expression in the hypothalamus (**c**) and hippocampus (**d**) showed that a significant decrease in *Tnf-α* mRNA expression was observed in astrocytes from α_2_^+/G301R^ mice (*n* = 3) after LPS treatment compared with α_2_^+/+^ astrocytes (*n* = 3). All data are presented as the mean ± SEM. *p < 0.05, **p < 0.01, ***p < 0.001 (Kruskal–Wallis test followed by Dunn’s post hoc test).

It is well known that LPS acts as an agonist of TLR4 to induce neuroinflammation and the expression of proinflammatory cytokines. To determine whether the LPS-induced expression of proinflammatory cytokines is associated with a potential effect of α_2_ haploinsufficiency in the TLR4 induction in hypothalamus and hippocampus, we investigated the expression levels of *Tlr4* in the hypothalamus and hippocampus by RT-qPCR. No difference in *Tlr4* gene transcription between the α_2_^+/+^ and α_2_^+/G301R^ mice in either the PBS or LPS treatment group was observed in the hypothalamus or hippocampus (Supplementary Fig. 6). However, as the α_2_ isoform is specifically expressed in astrocytes, we speculated that α_2_ haploinsufficiency would cause astrocytes from the α_2_^+/G301R mice to^ have lower levels of α_2-_Na^+^/K^+^-ATPase, and therefore, an effect on *Trl4* mRNA expression may be noted only in astrocytes and not in samples containing all cells from the hypothalamus or hippocampus. We therefore measured *Tlr4* expression in astrocytes isolated by bead sedimentation using an antibody against astrocyte cell surface antigen-2 (ACSA-2)^45^. In agreement with the established LPS-induced upregulation of TLR4, *Tlr4* expression was significantly increased in astrocytes from both the hypothalamus (blue in Fig. 5a) and hippocampus (red in Fig. 5b) in the α_2_^+/+^ mice after LPS treatment compared with PBS treatment. *Tlr4* expression in the astrocytes from the α_2_^+/G301R^ mice was comparable with that in the astrocytes from the α_2_^+/+^ mice treated with PBS; however, no increase in *Tlr4* expression was observed in the hypothalamus (Fig. 5a) or hippocampus (Fig. 5b) of the α_2_^+/G301R^ mice after LPS treatment, suggesting that the α_2_ isoform is required for LPS-mediated effect on *Tlr4* expression in astrocytes during the acute LPS-induced neuroinflammation.

Ligand-induced receptor dimerization is thought to initiate signal transduction through TLRs, which leads to the recruitment of signalling adaptor proteins and the regulation of TNF-α mRNA translation^46,47^. Therefore, we hypothesized that the reduced expression of Tlr4 in astrocytes from α_2_^+/G301R^ mice could explain the reduced Tnf-α expression in hypothalamus and hippocampus of α_2_^+/G301R^ mice. As the amount of astrocytes isolated form these brain areas are too low to measure TNF protein levels, we measured *Tnf* mRNA expression to investigate this, in astrocytes isolated from either the hypothalamus or the hippocampus of α_2_^+/G301R^ mice compared to those isolated from α_2_^+/+^ (Fig. 5c,d), in line with the results of *Tlr4* expression. In the hypothalamus there is a significant increase after LPS, though smaller than in α_2_^+/+^ astrocytes and in the hippocampus there is a significant reduction.

Our results demonstrated an increase in the proportion of *Tnf/β-actin* mRNA in α_2_^+/+^ mice astrocytes in the hypothalamus (Fig. 5c) and hippocampus (Fig. 5d) after treatment with LPS. Interestingly, we observed a reduction in *Tnf* transcription in hippocampal astrocytes from α_2_^+/G301R^ mice after the challenge with LPS (Fig. 5d), supporting the results that astrocytes in these areas of the brain are differentially affected by α_2_ haploinsufficiency.

Together, these results show that the lack of the TNF-α response in the α_2_^+/G301R^ mice is partly due to the lack of α_2_Na^+^/K^+^-ATPase expressing astrocytes that can sense LPS through induced *Tlr4* expression.

Equal cytosolic levels of p65 in LPS-treated α_2_^+/G301R^ hippocampus.

**Figure 6:**
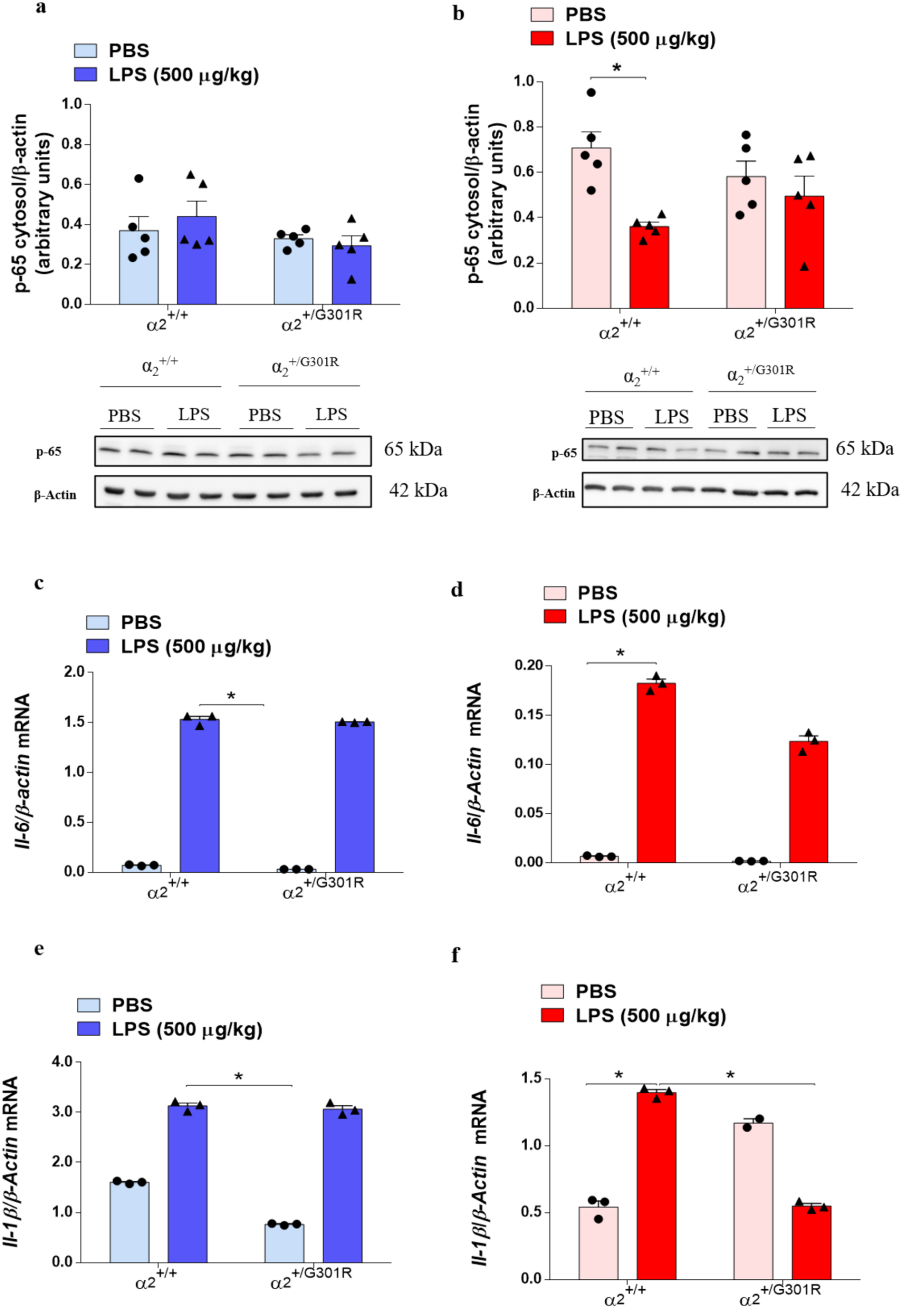
LPS decreases the cytoplasmic fraction of of NFκB in the hippocampus. (**a**,**b**) Western blotting densitometric analysis (arbitrary units) and representative Western blots for p65 (NFκB) and β-actin in the hypothalamus (**a**) (blue bars) (*n* = 5 mice/group) and hippocampus (**b**) (red bars) of the α_2_^+/+^ (*n* = 5) and α_2_^+/G301R^ (*n* = 5) animals 4 h after LPS treatment. All data are presented as the mean ± SEM. *p < 0.05 (Kruskal–Wallis test followed by Dunn’s post hoc test). RT-qPCR analysis of (**c**,**d**) *Il-6* and (**e**,**f**) *Il-1β* relative to *β*-actin expression in astrocytes isolated from the hypothalamus (**c**,**e**) and hippocampus (**d**,**f**) showed significant increases after LPS treatment for all except *Il-1β* in hippocampus of α_2_^+/G301R^ mice (*n* = 3 for both groups). All data are presented as the mean ± SEM. *p < 0.05, **p < 0.01, ***p < 0.001 (Kruskal–Wallis test followed by Dunn’s post hoc test).

LPS binding to TLR4 initiates an intracellular signaling cascade that results in NFκB activation. The activation of the transcription factor NFκB is initiated upon a nuclear translocation. NFκB is an important transcriptional regulator of neuroinflammation that is activated by LPS and/or proinflammatory cytokines, such as TNF-α, which trigger intracellular localization of the NFκB complex. Therefore, next to further dissect the mechanisms of α_2_ isoform-mediated regulation of LPS response, we evaluated the nuclear translocation of NFκB in the hypothalamus and hippocampus of LPS-treated α_2_^+/G301R^ mice. We examined nuclear protein levels via Western blotting using an antibody that recognize RelA (p65), a component of the NFκB complex. No significant changes were observed in cytoplasmic p65 levels in the hypothalamus (Fig. 6a, Supplementary Fig. 7), but there was a reduction in the hippocampus (Fig. 6b, Supplementary Fig. 8) of α_2_^+/+^ mice and the α_2_^+/G301R^ mice treated with PBS or LPS. In the nucleus, active NFκB promotes the transcription of NFκB-responsive genes^48,49^, such as IL-1β and IL-6. Therefore, next we measured *Il-6* and *Il-1β* mRNA levels in astrocytes isolated from both the α_2_^+/+^ and α_2_^+/G301R^ mice subjected to PBS or LPS treatment. These results showed that *Il-6* mRNA was upregulated in astrocytes isolated from the hypothalamus (Fig. 6c) and hippocampus (Fig. 6d) of α_2_^+/+^ and α_2_^+/G301R^ mice after LPS treatment. *Il-1β* mRNA was significantly upregulated in astrocytes isolated from the hypothalamus (Fig. 6e) and the hippocampus of α_2_^+/+^ mice after LPS treatment (Fig. 6f). However, *Il-1β* mRNA was significantly reduced in hippocampal astrocytes of α_2_^+/G301R^ mice after LPS treatment (Fig. 6f). Comparing the mRNA expression of *Il-1b* and *Il-6* in whole hypothalamus (Fig. 4a–c) and Hippocampus (Fig. 4d–f), there is an overall agreement that the effect is very local to the astrocytes, as the specific cells that is subjected to α_2_ haploinsufficiency.

This suggest that despite no significant nuclear translocation of NFκB was observed after LPS treatment in the α_2_^+/G301R^ mice, there is however, a significant induction of *Il-1b* and *Il-6* mRNA, suggesting either that the nuclear translocation assay needs to be performed in astrocyte populations, or supplementary mechanisms mediate the expression of the measured cytokines.

### Hippocampal astrocytes from α_2_^+/G301R^ mice express higher levels of *Nrf2* than α_2_^+/+^ cells

**Figure 7:**
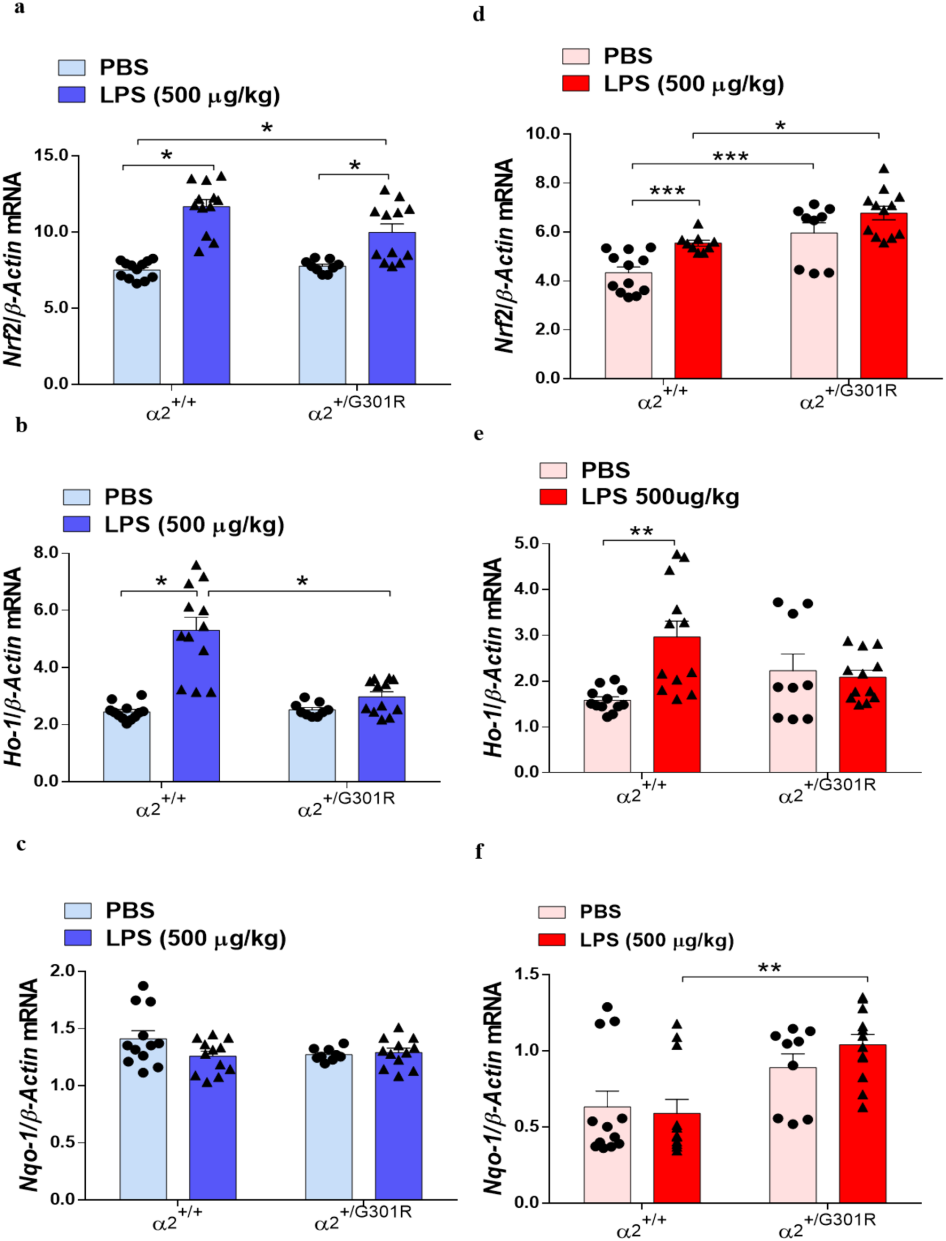
The α_2_ G301R mutation increases expression of the antioxidant enzyme NRF2-encoding transcript. (**a**–**f**) RT-qPCR mRNA expression analysis for *Nrf2* (**a**,**b**); *Ho-1* (**c**,**d**); *Nqo-1* (**e**,**f**) in the hypothalamus (blue bars) and hippocampus (red bars). All data are the mean ± SEM of three individual experiments. *p < 0.05, *p < 0.01, and ***p < 0.001 (two-way ANOVA followed by Tukey’s post hoc test, *n* = 3–4 mice/group).

The transcription factor NRF2 is an important regulator of the inflammatory response^50,51^. It regulates the expression of phase II detoxifying enzymes, including NADPH, NAD(P)H quinone oxidoreductase 1, glutathione peroxidase, ferritin hem oxygenase-1 (HO-1), and other genes that combat injury and inflammation^52,53^. Interestingly, p65 has been shown to activate NRF2 by sequestering the NRF2-regulator protein KEAP1 and thus leads to the transactivation of NRF2-dependent genes^54–57^. Therefore, next we investigated whether the NRF2 pathway is involved in the reduced LPS-induced neuroinflammation in the α_2_^+/G301R^ mice by measuring *Nrf2* expression in astrocytes of from hypothalamus and hippocampus. Interestingly, LPS induced *Nrf2* upregulation in in hypothalamic (Fig. 7a) and hippocampal (Fig. 7b) astrocytes from the α_2_^+/G301R^ and α_2_^+/+^ mice. Compared with the α_2_^+/+^ mice, the α_2_^+/G301R^ mice constitutively expressed higher levels of *Nrf2* in the hippocampus (Fig. 7b) suggesting that the astrocytes are affected by α_2_ haploinsufficiency even under naïve conditions.

In conclusion, these results show that *Nrf2* mRNA transcription is activated in the α_2_^+/G301R^ mice and of relevance to LPS-induced response.

To study if the observed increase in *Nrf2* mRNA expression in LPS-treated hypothalamus and hippocampus of the α_2_^+/G301R^ and α_2_^+/+^ mice resulted in subsequent expression of NRF2-responsive genes, we measured the expression of the NRF2-responsive genes *Ho-1* and *Nqo1*^58^ in astrocytes from the hypothalamus and hippocampus of α_2_^+/+^ and α_2_^+/G301R^ mice.

LPS treatment resulted in increased *Ho-1* expression in astrocytes from the hypothalamus (Fig. 7c) and hippocampus (Fig. 7d) of α_2_^+/+^ mice compared to that in PBS-treated mice.

However, LPS did not lead to an increase in *Ho-1* expression in the hypothalamus or in the hippocampus of α_2_^+/G301R^ mice (Fig. 7c,d). No differences in *Nqo1* expression between the hypothalamic astrocytes from the α_2_^+/G301R^ and α_2_^+/+^ mice were observed after LPS treatment (Fig. 7e,f), while *Nqo1* expression was increased in the hippocampal astrocytes from the of α_2_^+/G301R^ mice 4 h after treatment with LPS (Fig. 7f).

Combined this indicate that *Nfr2* expression increases after LPS treatment in both hypothalamus or in the hippocampus of α_2_^+/+^ mice and α_2_^+/G301R^ mice. Of NRF2-responsive genes, *Ho-1* mRNA appears reduced in astrocytes from hypothalamus of α_2_^+/G301R^ mice compared to α_2_^+/+^ mice, however, only a difference was observed for *Nqp-1* mRNA in astrocytes from hippocampus of α_2_^+/G301R^ mice compared to α_2_^+/+^ mice.

### α_2_ haploinsufficiency correlate directly with the increased expression of *Cox2 transcripts* in hippocampal astrocytes

**Figure 8:**
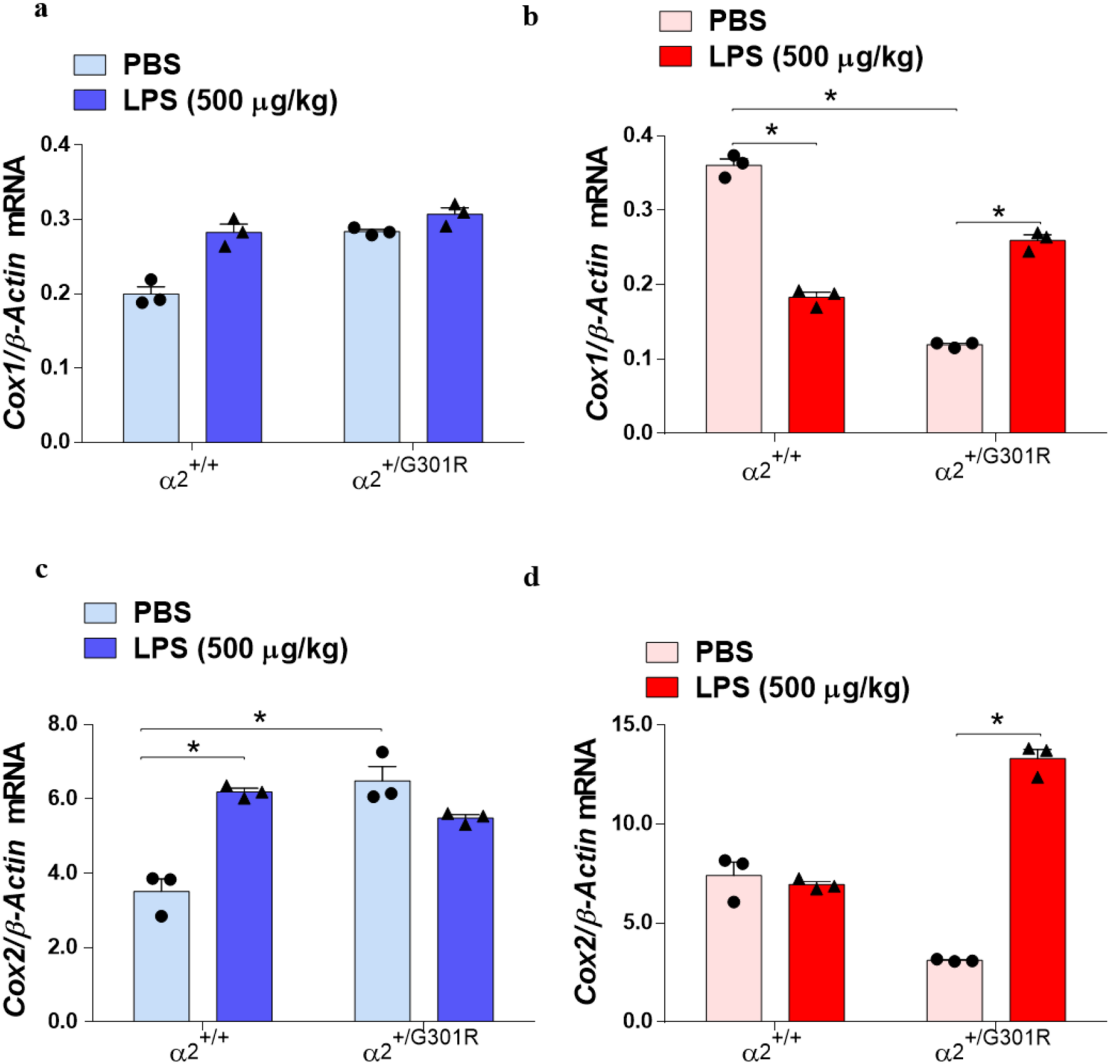
α_2_ haploinsufficiency alters *Cox1 and Cox2* gene expression in astrocytes from the hypothalamus and hippocampus of α_2_^+/+^ mice and α_2_^+/G301R^ mice. (**a**–**d**). qPCR mRNA expression analysis for *Cox1* (**a**,**b**); *Cox2* (**c**,**d**). All data are the mean ± SEM of three individual experiments. *p < 0.05, *p < 0.01, and ***p < 0.001 (Kruskal–Wallis test followed by Dunn’s post hoc test, *n* = 3 mice/group).

LPS-mediated inflammation triggers the expression level of *COX genes*, major inflammatory factors^59^. To determine the role of the α_2_ isoform in LPS-induced *Cox1* and *Cox2* expression, we performed RT-qPCR analysis on astrocytes isolated from the hypothalamus and hippocampus of the PBS- and LPS-treated α_2_^+/+^ and α_2_^+/G301R^ mice. We found that there was no difference in the expression of the *Cox1* gene in the hypothalamic astrocytes of all groups evaluated (Fig. 8a). In α_2_^+/G301R^ astrocytes from the hippocampus, we noted that *Cox1* gene expression was significantly increased after LPS treatment, in contrast to PBS-treated α_2_^+/G301R^ hippocampal astrocytes (Fig. 8b).

The *Cox2* mRNA expression in α_2_^+/+^ mice astrocytes was upregulated in hypothalamus after the challenge with LPS (Fig. 8c), but unchanged in hippocampus (Fig. 8d).

In astrocytes from the hypothalamus, *Cox2* mRNA was significantly elevated in PBS-treated α_2_^+/G301R^ mice, with no further induction was noted in astrocytes from the α_2_^+/G301R^ mice upon LPS treatment (Fig. 8c). In astrocytes from the hippocampus, the *Cox2* levels in the α_2_^+/+^mice were not altered (Fig. 8d). In contrast, *Cox2* mRNA expression was significant upregulated in LPS-treated hippocampal astrocytes from the α_2_^+/G301R^ mice (Fig. 8d).

Overall, this correlates very well with the tendency of increased nuclear levels of p65, which suggests increased NFκB activity in astrocytes from the hippocampus (Fig. 6b) and is responsible for high *Cox2* expression^59^, as noted in the same cells (Fig. 8b).

### LPS-induced memory impairment and anxiety-like behaviours are not significantly altered in α_2_^+/G301R^ mice compared with WT littermates

Because long-lasting effects in memory and behavior have been observed following LPS administration (reviewed in^60^), we wished to examine if the α_2_^+/G301R^ mice were susceptible for behavior changes upon LPS administration.

**Figure 9:**
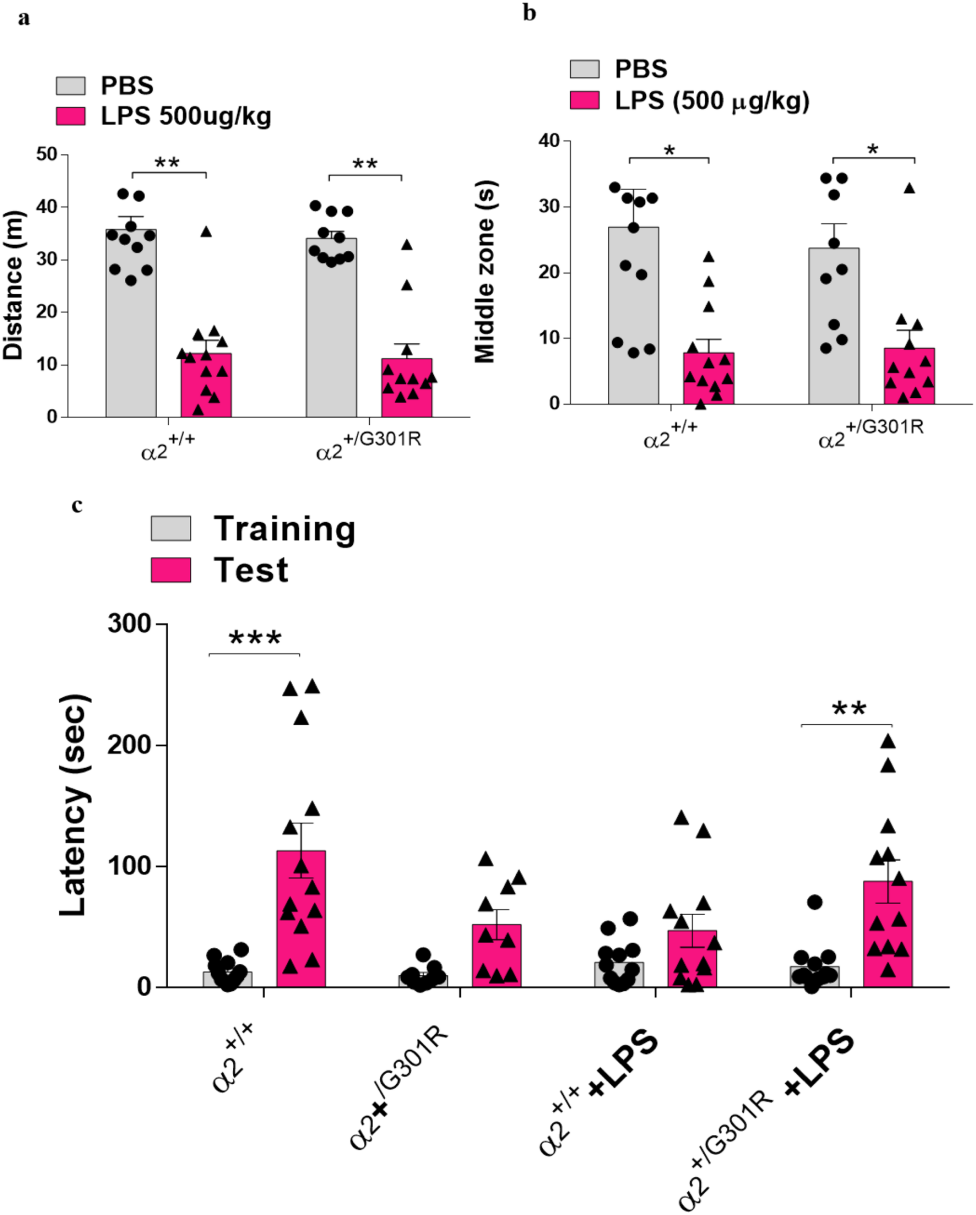
α_2_Na^+^/K^+^-ATPase isoform does not alter the memory impairment or anxiety that is induced by LPS. (**a**,**b**) Results of analysis of behaviour in the open-field test 4 h after LPS injection showing the total distance travelled (**a**) and time spent in the centre of the open field (**b**) (*n* = 10–12). (**c**) The passive avoidance test results (*n* = 10–12). The training and test were performed 24 and 72 h after LPS injection, respectively. All data are presented as the mean ± SEM. *p < 0.05, **p < 0.01, and ***p < 0.001 (Kruskal–Wallis test followed by Dunn’s post hoc test).

To assess whether the α_2_ isoform is involved in the cognitive and affective LPS-mediated effects, first, we examined the locomotor activity of the α_2_^+/G301R^ mice versus the α_2_^+/+^ mice treated with saline or LPS using the open field test 4 h after PBS or LPS administration. LPS induced a reduction in locomotion and anxiety-like behaviours in both the α_2_^+/+^ and α_2_^+/G301R^ mice (Fig. 9a,b). There was no difference in the total distance travelled (*P* = 0.5806) or the time spent in the middle zone of the arena (*P* = 0.7457) by LPS-treated α_2_^+/+^ and α_2_^+/G301R^ mice. Similarly, no differences were observed between the two types of mice after PBS treatment.

Next, we investigated whether α_2_ isoform haploinsufficiency can influence LPS-induced memory impairment. To this end, we assessed fear memory using the passive avoidance test that measures the latency to enter a dark environment in which an aversive stimulus (foot shock) has been previously experienced using a light–dark box paradigm. All four groups (PBS- and LPS-treated α_2_^+/+^ and α_2_^+/G301R^ mice) showed similar baseline latencies to enter the dark chamber in the training stage (Supplementary Fig. 9). We evaluated the latency for entry to the dark side of the box before the challenge with LPS and observed that the isoform α2-Na,K-ATPase mutation does not interfere with the time of entry to the chamber in the training stage (Supplementary Fig. 9a). Despite the increased latency time for α_2_^+/G301R^ mice, in the test, after treatment with LPS, it showed that α2^+/G301R^ mice are less cognitively affected than α_2_^+/+^ animals, after treatment with LPS (Fig. 9c), and we did not observe differences in latency between the mutated animals treated with PBS or LPS (Supplementary Fig. 9b).

The α_2_^+/+^ and α_2_^+/G301R^ mice treated with PBS showed a significant increase in latencies in the probe stage, compared to LPS-treated α_2_^+/+^ mice (Fig. 9c). The increased time in latency was found significant for α_2_^+/G301R^ mice, in the test, after LPS treatment, suggesting that the α_2_^+/G301R^ mice are less cognitive affected than their littermates, after LPS treatment.

Collectively, our results suggest that the reduction of the α_2_ isoform does not interfere with the LPS-induced decrease in locomotor activity, increase in anxiety behaviours, or cognitive impairment. Activation of the immune system by LPS leads to production and release of proinflammatory cytokines such as TNF-α, IL-1β that act on the periphery and central nervous system leading to symptoms such as immobility and/or lethargy, piloerection, drowsiness, and ptosis, this symptoms are knowing as sickness behavior^61^, which may be accompanied by physiological changes such as hypothermia. The sickness behavior reduces with the process of resolving inflammation, but motivational deficits may last longer, such as anxiety. Given this, we can infer that the reduction in α_2_Na^+^/K^+^-ATPase activity interferes with sickness behavior, since we observed a less locomotion and hypothermia (Fig. 1a,b). However, there was no motivational change as observed by the open field test (Fig. 9a,b).

## Discussion

Neuroinflammation is a critical factor in neurodegenerative diseases, including Parkinson’s disease and Alzheimer’s disease. We used a gene modified mouse with a knock-in mutation to investigate the role of the vital membrane ion pump Na^+^/K^+^-ATPase in mediating neuroinflammation. In recent years, there has been increasing evidence that the pump, the Na^+^/K^+^-ATPase, serve not only to maintain the electrochemical gradient across the cell’s plasma membrane, but are highly involved in regulating intracellular signal cascades.

**Figure 10:**
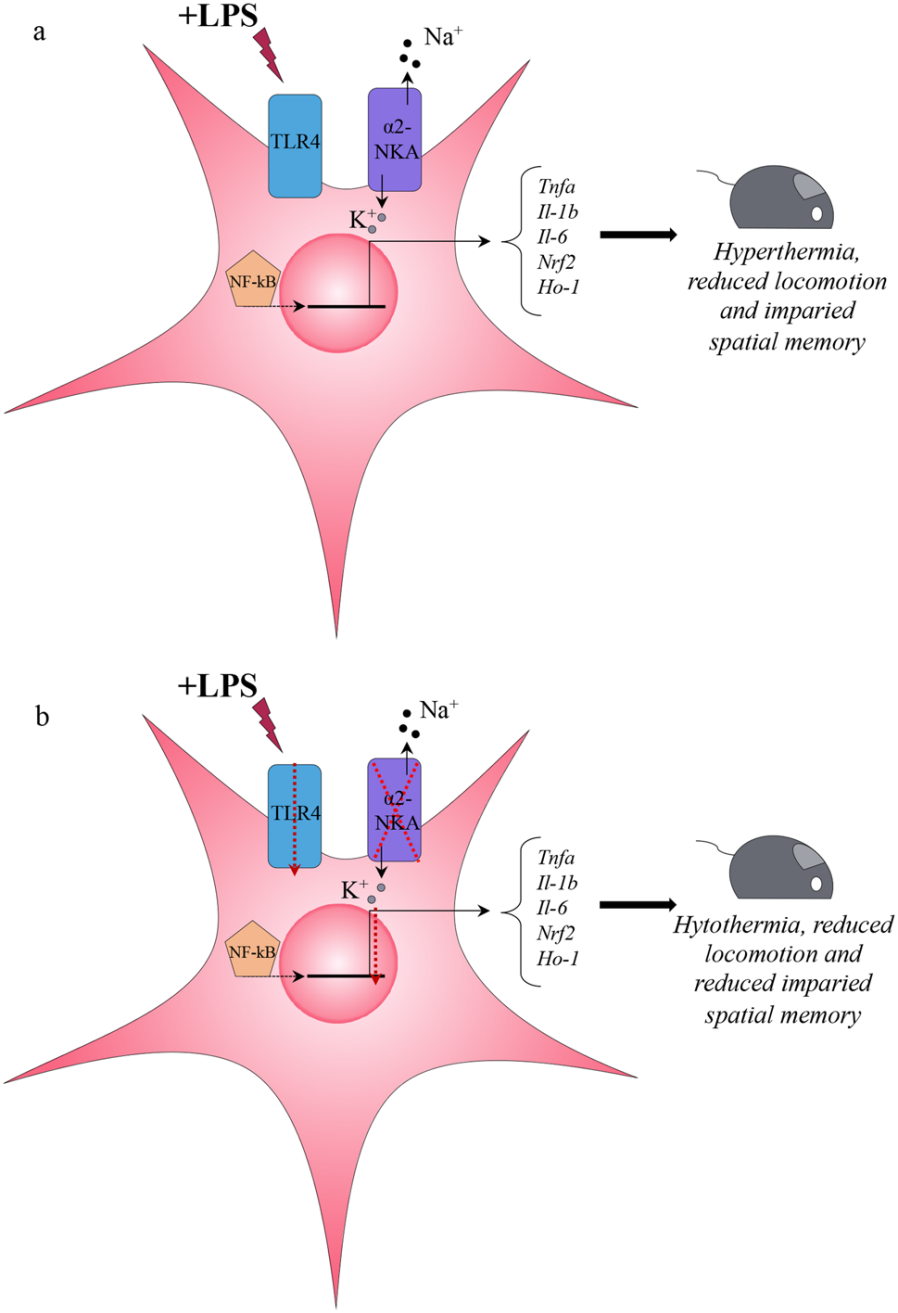
Schematic drawing of the proposed action upon LPS treatment in astrocytes expressing the α_2_Na^+/^K^+^-ATPase (noted α_2_NKA in the figure). (**a**) In astrocytes with normal α_2_Na^+/^K^+^-ATPase function, the intracellular responses to LPS will lead to an increasement of several inflammatory genes and cause LPS-related phenotypes in the mouse, including hypothermia, reduced locomotion and impaired spatial memory. (**b**) In astrocytes with α_2_Na^+/^K^+^-ATPase haploinsufficiency, the intracellular responses to LPS will lead to a significantly reduced expression of several inflammatory genes and compromise LPS-related phenotypes in the mouse, leading to less hypothermia, reduced locomotion and reduced impaired spatial memory.

We report that mice that are haploinsufficient for the astrocyte-enriched α_2_Na^+^/K^+^-ATPase isoform (α_2_^+/G301R^ mice) have a reduced proinflammatory response to LPS, accompanied by a reduced hypothermic reaction. Following administration of LPS, the gene expression of *TNF-α*, *IL-1β*, and *IL-6* was reduced in the hypothalamus and hippocampus from α_2_^+/G301R^ mice compared to WT littermates (Fig. 10).

The central nervous system regulates systemic inflammatory responses to endotoxin through humoral mechanisms via activation of the hypothalamic–pituitary–adrenal pathway, as well as through the activation of efferent vagal nerve pathways by reducing the production of pro-inflammatory cytokines produced by macrophages^62,63^. Therefore, we can assume that neuroinflammation reduction observed in the α_2_^+/G301R^ animals could be due to a possible interference in the neuroimmune pathways, however studies must be carried out to understand this interference. Furthermore, activation of the immune system by LPS leads to production and release of proinflammatory cytokines such as TNF-α, IL-1β that act on the periphery and central nervous system leading to symptoms such as immobility and/or lethargy, piloerection, drowsiness, and ptosis, this symptoms are knowing as sickness behavior, which may be accompanied by physiological changes such as hypothermia. The sickness behavior reduces with the process of resolving inflammation, but motivational deficits may last longer, such as anxiety. Given this, we can infer that the reduction in α_2_Na^+^/K^+^-ATPase activity interferes with sickness behavior, since we observed a less locomotion and hypothermia (Fig. 1a,b). However, there was no motivational change as observed by the open field test (Fig. 9a,b). The α_2_^+/G301R^ mice experienced increased expression of the gene encoding an antioxidant enzyme, *Nrf2*, in hippocampal astrocytes.

Here, we demonstrated by complementary in vitro and in vivo experimentation that the production of proinflammatory cytokines upon LPS stimuli is highly dependent on the astrocyte-specific α_2_ isoform of Na^+^/K^+^-ATPase. α_2_ Haploinsufficiency differentially altered the production of TNF-α, IL-6, and IL-1 in LPS-treated astrocytes. This appeared to be associated with a reduced *Tlr4* expression.

The LPS-induced immune response in hypothalamus and hippocampus is different and may very well associated differential pathway activation promoted by TLR4, such as NFkB and MAPK, but may also involve other pathways^64^, yet to be elucidated. Interestingly, this caused a decrease in the hypothermic response in α_2_^+/G301R^ mice compared with their α_2_^+/+^ littermates, which might be associated with a reduced expression of *Nrf2*, a mediator of inflammatory pathways^50,51^, in the hypothalamus in response to LPS. The LPS induced impairment of fear memory and anxiety-like behaviours observed in α_2_^+/+^ mice was not affected by α_2_ isoform haploinsufficiency.

Hypothermia is a thermoregulatory response to systemic inflammation that can be induced in rodents in response to systemic LPS challenge^65,66^. Although the molecular mechanisms of LPS-induced hypothermia are still poorly understood, the participation of cytokines has been observed in the development of hypothermia^67^. The reduced cytokine levels we found in α_2_^+/G301R^ mice could explain the lower hypothermic response in these animals following LPS treatment.

Previous findings have shown that ouabain, which acts as a Na^+^/K^+^-ATPase inhibitor, can protect motor neurons from mutant SOD1–induced astrocyte degeneration and reduce the neuroinflammation resulting from LPS^36,44^. In addition, haploinsufficiency-causing Na^+^/K^+^-ATPase α_2_ isoform G301R mutation decreases lesion volume and improves functional outcomes after acute spinal cord injury in mice^35^. Furthermore, silencing of α_2-_Na^+^/K^+^-ATPase reduces LPS-induced inflammation in glial cells^39^.

We previously studied TNF, IL-6 and IL-10 production in the spinal cord of α_2_^+/G301R^ and α_2_^+/+^ mice with spinal cord injury^35^. We found upregulation of these cytokines in mice with spinal cord injury compared to uninjured mice but we observed no significant differences between the two genotypes; only IL-1β appeared to display a tendency to be upregulated^35^, but it is important to consider that IL-1β is produced as precursor molecules, pro-IL-1β, which is cleaved in its active form by a caspase family cysteine protease, the IL-1β converting enzyme (ICE)^68^. Although there are different mechanisms proposed for post-translational processing of IL-1β, there is still no definite mechanism. Our data show that LPS challenge promoted a serum increase of IL-1β that was not reversed in a_2_ gene modified animals, thus suggesting that deficiency in a_2_ activity does not interfere with the release of IL-1β in its mature form. Combined, these previous and our new findings support a role for the α_2_ isoform of Na^+^/K^+^-ATPase in LPS-induced neuroinflammation, by means of mediating cytokine expression.

LPS induces an innate immune response and the production of various proinflammatory cytokines via TLR4 activation. In view of this, we hypothesized and confirmed that α_2_Na^+^/K^+^-ATPase haploinsufficiency prevents an increase in *Tlr4* expression after LPS exposure in astrocytes of the hypothalamus and hippocampus.

Recently, a study showed that neuronal activity regulates the astrocytic signalling of the nuclear master transcription factor NRF2 through the secretion of glutamate and other soluble factors^69^. Thus, we hypothesized that α_2_^+/G301R^ mice constitutively exhibit an increase in NRF2 activity that protects these animals from LPS-induced neuroinflammation, as NRF2 is capable of negatively regulating NFkB activity and the consequent reduced expression of pro-inflammatory cytokines. Confirming this hypothesis, we found that *Nrf2* expression was upregulated in hippocampus, and *Nrf2* expression was further enhanced upon LPS treatment, suggesting this as one mechanism that reduce NFkB activity in cells haploinsufficient for the α_2_ isoform.

Moreover, the NRF2-responsive antioxidant factor *Nqo-1* mRNA was upregulated in the hippocampus in α_2_^+/G301R^ mice in the absence of inflammatory stimulation, and only marginally increased upon LPS treatment, suggesting the involvement of NRF2 activation in the reduced neuroinflammatory response to LPS exposure in α_2_^+/G301R^ mice. This is in line with the fact that NQO-1 exhibits anti-inflammatory activity by inhibiting the induction of TNF and IL-1β expression by LPS in human monocytes^51,70^.

Peripheral LPS administration promotes NFκB activation in various regions of the central nervous system, leading to the stimulation of proinflammatory cytokines^71^. Our results confirmed that LPS-induced activation of NFĸB occurred by measuring reduced levels of cytoplasmic p65, which increased proinflammatory genes within 4 h in the hippocampus of α_2_^+/+^ mice.

IL-1β has been associated with cognitive impairment during the inflammatory process, and the intra-hippocampal administration of IL-1β induces impaired memory consolidation and reconsolidation in rats^72^. Although our results showed a reduction in IL-1β expression in the hippocampus 4 h after LPS administration in α_2_^+/G301R^ animals, we observed that that α_2_ haploinsufficiency does not affect the LPS-induced effect on memory impairment and anxiety.

While the present study addressed the regulation of cytokines in relation to the Na^+^/K^+^-ATPase in astrocytes from hypothalamus and hippocampus, future studies must delineate the mechanisms in other brain structures as well as the α_2_ isoform is expression in astrocytes throughout the brain. Of immediate interest in this context is the synergy between signaling pathways that mediate the α_2_-dependent LPS-induced neuroinflammation. Studies have demonstrated the existence of an important interaction between adipocytes and macrophages for the amplification of the inflammatory response induced by LPS^73^, and knowing that adipocytes express the α_2_Na^+^/K^+^-ATPase isoform^74,75^, it is likely that the a2 isoform in adipocytes contributes to the reduction of circulating levels of cytokines, however, this remains to be explored.

In summary, this study provides an undescribed link between the α_2_Na^+^/K^+^-ATPase and inflammation signaling in vivo. Overall, regulation of the astrocyte α_2_ isoform represents a significant regulator of inflammatory responses in the brain, with makes the α_2_ isoform a likely candidate for depressing neuroinflammation, and perhaps also neurodegenerative conditions.

## Methods

### Experimental animals

Mice were cared for in accordance with the protocols and guidelines that The Danish Animal Inspectorate approved under the Ministry of Food and Agriculture, Denmark (J. No. 2013-15-2934-00815 to KLH). We confirm that all experimental protocols were approved by the Animal Facility and the veterinary surgeon at the Department of Biomedicine, Aarhus University, Denmark.

Our mice were bred on a C57/BL6JRj (Janvier) background. All animals were housed under a reverse light/dark cycle to prevent daytime experiments from interfering with their normal sleep cycles.

All experiments were performed on 8- to 12-week-old α_2_^+/G301R^ mice and α_2_^+/+^ mice, and all mice used are summarized in Table 1.

**Table 1:** Distribution of genotype and sex of animals used.

Sex	Genotype	Biochemical	Behavior	Macrophages culture	Body temperature	Total
Female	α_2_^+/+^	26	23	3	11	63
Female	α_2_^+/G301R^	12	22	3	5	42
Male	α_2_^+/+^	16	10	3	9	38
Male	α_2_^+/G301R^	14	11	4	10	39

The animals were housed in ventilated cages with 1–3 cage mates under a 12-h light/dark cycle and controlled temperature and humidity and with free access to food and water. The animals were treated with 500 µg/kg LPS (#L2630, O11:B4) (Sigma-Aldrich, St. Louis, MO) or PBS. Four hours after LPS administration, the mice were anaesthetized by isoflurane inhalation and euthanized by decapitation, and the brains were immediately removed and immersed in cold PBS. The hippocampus and hypothalamus were rapidly dissected, quickly immersed in liquid nitrogen, and stored at − 80 °C for later use.

### Genotyping

Heterozygous α_2_^+/G301R^ mice and α_2_^+/+^ mice were genotyped^33^ via high resolution melt analysis (Roche LightCyclerR 96 Real-Time PCR System) using the following primers: F, 5′-ggatgagggacagaacgaag and R, 5′-catggagatcgagcatttca (Sigma-Aldrich).

### Cell culture procedures

To isolate bone marrow–derived macrophages (BMDMs), bone marrow was isolated from the femurs and tibias of 9-week-old WT and α_2_^+/G301R^ mice, and the cells cultured in Roswell Park Memorial Institute (RPMI) 1640 medium (Sigma-Aldrich, St. Louis, MO) supplemented with 10% heat-inactivated foetal bovine serum (FBS) (Sigma-Aldrich, St. Louis, MO), 100 U/mL penicillin/streptomycin, and 20% of L929 conditioned media (Sigma-Aldrich, St. Louis, MO) at 37 °C in 5% CO_2_^76,77^. On day 2, 5 mL of supplemented RPMI 1640 medium and 40% L929 conditioned medium was added. On day 4, the non-adherent cells were removed from the flask, the media was replaced, and the remaining adherent cells were maintained in culture for 6 days in 20% L929 conditioned medium. On day 7, the cells were transferred to 24-well plates (4 × 10^5^ cells per well) and cultured for 4 h before use. The cells were treated with PBS or LPS (100 ng/mL) for 1, 2, 4, and 6 h. Supernatant samples were collected for the analysis of TNF-α via ELISA.

### Body temperature

The rectal body temperature was measured using a rectal probe (TFN 530 SMP Thermometer, Ebro) at the time of the first injection and 4 h after the injection of LPS or PBS.

### Measurement of cytokine levels

Four hours after LPS or PBS injection, blood was collected in 15-mL conical tubes and centrifuged at 3,000 rpm for 10 min to obtain the serum. The concentrations of TNF-α (#88-7324), IL-1β (#MLB00C), IL-6 (#M6000B), and IFN-γ (#MIF00) were measured by mouse-specific sandwich ELISA according to the manufacturer’s instructions (eBioScience, Santa Clara, California, USA and R&D Systems, Minneapolis, USA)^78^. Briefly, samples were added to coated microwells with antibodies against TNF-α, IL-1β, IL-6, and IFN-γ along with a biotin-conjugated antibody (horseradish peroxidases; Mouse IL-1β Conjugate # 893830), polyclonal antibody specific for mouse IL-1β conjugated to horseradish peroxidase with preservatives (R&D Systems), Mouse IL-6 Conjugate # 892665, polyclonal antibody against mouse IL-6 conjugated to horseradish peroxidase with preservatives (R&D Systems), Mouse IFN-γ Conjugate (# 892666, polyclonal antibody specific for mouse IFN-γ conjugated to horseradish peroxidase with preservatives (R&D Systems). The Mouse TNF-α Conjugate (#88-7324), (eBioScience). The plate was incubated for 2 h at room temperature. The wells were washed, streptavidin-HRP was added to the entire plate, and the plate was incubated for 1 h at room temperature. Subsequently, the wells were washed and then incubated with TMB substrate (Thermo Fisher Scientific, Roskilde, Denmark) solution for 30 min while being protected from light. The reaction was stopped with a stop solution, and the absorbance was measured using a spectrophotometer at 450 nm. The concentrations of the cytokines were measured based on the standard curve.

### Protein extraction and immunoblot analysis

The hippocampus and hypothalamus were isolated according to the protocol of the CelLytic NuCLEAR Extraction Kit (Sigma-Aldrich, St. Louis, MO). In brief, the tissues were homogenized in lysis buffer containing 10 mM HEPES (pH 7.9) with 1.5 mM MgCl_2_, 10 mM KCl, 0.1 M dithiothreitol (DTT) solution and a protease inhibitor cocktail, and they were centrifuged at 10,000×*g* for 20 min. The supernatant representing the cytosolic fraction was transferred to a new tube. The pellet was resuspended in an extraction buffer containing 1.5 μL 0.1 M DTT and 1.5 μL protease inhibitor cocktail. The solution was allowed to stand on ice for 30 min with shaking at brief intervals followed by centrifugation at 20,000×*g* for 5 min. The supernatant, which contained the nuclear protein fraction, was transferred to a clean chilled tube. The proteins from the cytosolic fractions of the hippocampus and hypothalamus (20 μg) were separated by size by 10% sodium dodecyl sulfate polyacrylamide gel electrophoresis. The proteins were blotted onto a nitrocellulose membrane (Pharmacia-Amersham, Amersham, UK) and incubated with anti-α1 (1:500) (a6f.-c, Developmental Studies Hybridoma Bank, USA), anti-α2 (1:5,000) (07674, EMD Millipore, USA), anti-p65 (1:1,000) (CST-8242T, Cell Signalling), and actin (1:1,000) (A2066, Sigma-Aldrich,) primary antibodies overnight at 4 °C^33^. The secondary antibodies included horseradish peroxidase-conjugated pig anti-rabbit and pig anti-mouse (1:2,000) (Dako, Glostrup, Denmark) antibodies. The proteins recognized by the antibodies were revealed via an Amersham ECL Western Blotting Detection Kit, following the instructions of the manufacturer (GE Healthcare, Buckinghamshire, UK). To standardize and quantify the immunoblots, we used the photo documentation system of the LAS 3000 imager (Fujifilm, Tokyo, Japan) and ImageJ software (US National Institutes of Health, Bethesda, MD; https://rsb.info.nih.gov/ij), respectively. Several exposure times were analysed to ensure the linearity of the band intensities. β-Actin was used as an internal control for the experiments. The results are expressed in relation to the intensity of β-actin.

### Astrocyte dissociation

The hippocampus and hypothalamus tissues were dissociated using the Neural Tissue Dissociation Kit—Postnatal Neurons (catalogue number 130094802), as described by the manufacturer. Briefly, the structures were weighed and transferred to a gentleMACS C tube containing 1960 μL of enzyme mix (50 μL of enzyme P + 1910 μL of buffer Z) and 45 μL of enzyme mix 2 (30 μL of buffer Y + 15 μL of enzyme A). Then, the tubes were connected to the gentleMACS Octo Dissociator with Heaters, and the gentleMACS 37C_NTDK_1 programme was used for tissue dissociation. Then, the samples were centrifuged briefly, and the cells were resuspended in D-PBS. The cell suspension was filtered with a 70-μm cell strainer (MACS SmartStrainer), which was washed with 10 mL of D-PBS supplemented with BSA (0.5%). Thereafter, the cell suspension was centrifuged at 300×*g* for 10 min at room temperature, and the cells were suspended in D-PBS supplemented with BSA (0.5%).

### Myelin debris removal

Prior to the isolation of astrocytes, we performed a debris-removal step using protocols from Miltenyi Biotec’s Myelin Removal Kit (catalogue number 130096733). Following dissociation, the cells were incubated for 15 min at 4 °C with Myelin Removal Beads II, following the ratio of 500 mg of brain tissue in 1,800 μL of buffer + 200 μL of myelin removal beads)^45^. The cells were washed with 0.5% BSA in PBS and centrifuged at 300×*g* for 5 min to remove any unbound spheres from the pellet. After that, the pellet was resuspended in 500 μL of buffer, the suspension was added to a LS column prepared in the MACSMidi magnetic cell separator, and the flow-through was collected. The column was further washed three times with 3 mL of buffer to ensure the removal of the unlabelled cells. The cells retained on the column were eluted in 5 mL of buffer. The flow-through was used in subsequent steps for astrocyte isolation.

### Isolation of astrocyte cells

The astrocytes were positively selected using the protocol of the Miltenyi Biotec Anti-ACSA-2 Kit (catalogue number 130097678). Up to 1 × 10^7^ dissociated cells were suspended in 80 μL of buffer (0.5% BSA in PBS) and incubated with 10 μL of FcR blocking buffer for 10 min at 4 °C, followed by incubation with 10 μL of ACSA-2 MicroBeads for 10 min at 4 °C. Then, the cells were washed with 1 mL of buffer (0.5% BSA in PBS) and centrifuged at 300×*g* for 10 min to remove the excess beads from the solution. After the removal of the lavage solution, the pellet was resuspended in 500 μL of buffer, and the suspension was added to a prepared LS column installed in the MACSMidi magnetic cell separator. The column was washed with 3 mL of buffer three successive times to remove the unlabelled cells. After the column was removed from the magnetic separator, the astrocytes were eluted in 5 mL of buffer. The number of cells was then determined, and total RNA was extracted.

### Reverse transcription quantitative PCR (RT-qPCR)

Total RNA was isolated from astrocytes isolated from astrocytes from the hippocampus and hypothalamus with the RNeasy Plus Mini Kit (Qiagen), according to the manufacturer’s instructions. Complementary deoxyribonucleic acid (cDNA) was generated from 500 ng total RNA using the PrimeScript RT Reagent Kit (Takara BIO INC). The *Tnfα*, *Il-1β*, *Il-6*, *Tlr4, Nrf2*, *Ho-1*, and *Nqo-1β*, *Cox1/2*, *Ptgds* and *β-actin* (our reference) gene expression levels were measured via quantitative PCR (qPCR) using the TaqMan gene expression assays (Thermo Fisher Scientific) noted below:

**Table:** 

Gene	Primer ID
*Tnf-α*	Mm00443258_m1
*Il-1β*	Mm00434228_m1
*Il-6*	Mm00446190_m1
*Tlr4*	Mm00445273_m1
*Nrf2*	Mm00477784_m1
*Ho-1*	Mm00516005_m1
*Nqo-1β*	Mm01253561_m1
*Cox1*	Mm04225243_g1
*Cox2*	Mm03294838_g1
*β-Actin*	Mm02619580_g1

Real-time PCR analysis was performed in triplicate, with each reaction including 20 ng of cDNA, 5 μL of 2 × LightCycler 480 Probed Master Mix. (Roche, Basel, Switzerland), 0.5 μL of TaqMan Gene Expression Assay, and nuclease-free water up to a final volume of 10 μL. The PCRs were run in the Roche LightCycler 96-well system with the following protocol: 2 s at 50 °C for uracil-DNA glycosylase enzyme activation, 10 min at 95 °C for DNA Polymerase activation, 45 cycles of 15 s at 95 °C for denaturation, and 1 min at 60 °C for annealing and extension followed by a final denaturation step of 95 °C for 5 min^79,80^. The triplicate expression values of each gene were set relative to the expression of the reference gene via the delta-delta-Ct method^81^. As a negative control, RT-PCRs with no template were used.

### Behavioural analysis

#### Open field test

The mice were placed in the centre of an open-field apparatus (50 × 50 cm) (Stoelting Europe; Dublin, Ireland) and monitored for 15 min using ANY-maze software V4.99 (Stoelting, USA)^82^. The system automatically recorded the total distance travelled (m) and the time (s) spent in the centre zone. Each mouse (n = 10–12 for each group) was tested once 4 h after PBS or LPS injection, and the open-field setup was cleaned with 70% ethanol and wiped with paper towels between each trial.

#### Passive avoidance test

The training was initiated on the acquisition day, 24 h after PBS or LPS injection. Each mouse was placed in a brightly lit compartment with an electronically controlled door leading into a dark compartment^82^. The latency (s) for the mouse to enter the dark compartment was recorded. Once in the dark compartment, the door closed, and the mouse received an electric shock (0.42 mA for 1 s). The test was performed 72 h after PBS or LPS injection, the mouse was reintroduced to the same brightly lit compartment, and the latency to enter the dark compartment was recorded as an indicator of memory for the shock.

### Statistical analysis

The qPCR results were analysed via the delta-delta-Ct method, according to Schmittgen and Livak (2008) and calculated using REST 2009 Software (Qiagen, Duesseldorf, Ger). Normality was assessed through the D’Agostino and Pearson omnibus normality test, and, for parametric analyses. Parametric analyses were conducted through two-way ANOVA followed by Tukey’s post-test. Non-parametric analyses were conducted through Kruskal–Wallis test followed by Dunn’s post hoc test. Differences were considered to be significant at p < 0.05, and all results are expressed as the mean ± standard error of the mean (SEM) of the indicated number of experiments. All analyses were performed using the Prism 6 software package (GraphPad Software, San Diego, CA, USA).

## Supplementary information

Supplementary Figures.

Supplementary Video.

## Data Availability

We confirm that all relevant data from this study are available from the corresponding author upon request.
