# Supplementary material for: The α2 Na+/K+-ATPase isoform mediates LPS-induced neuroinflammation
Source: Sci Rep. 2020 Aug 25;10:14180. doi: 10.1038/s41598-020-71027-5 (PMC7447643; doi:10.1038/s41598-020-71027-5)
Supplement: Supplementary file 1 — Supplementary Figures. [file 41598_2020_71027_MOESM1_ESM.docx]

The α_2_ Na^+^/K^+^-ATPase isoform mediates LPS-induced neuroinflammation

Leite, JA^1,2,3^, Isaksen, TJ^1^; Heuck, A^1^, Scavone, C^2^, Lykke-Hartmann, K^1,4.5*^

^1^Department of Biomedicine, Aarhus University, Aarhus, Denmark

^2^Department of Pharmacology, Instituto de Ciências Biomédicas, Universidade de São Paulo, São Paulo, Brazil

^3^Department of Pharmacology, Instituto de Ciências Biomédicas, Universidade Federal de Goiás, Goiânia, Brazil

^4^Department of Clinical Medicine, DK-8000 Aarhus C, Aarhus University, Denmark

^5^Department of Clinical Genetics, DK-8200 Aarhus N, Aarhus University Hospital, Denmark

***Corresponding author**:

Lykke-Hartmann, Department of Biomedicine, Aarhus University, Aarhus, Denmark, [kly@biomed.au.dk](mailto:kly@biomed.au.dk)

**Supplementary Fig. 6**. The α_2_^+/G301R^ mice exhibited no differences in *Tlr4* expression in the hypothalamus (blue bars) and hippocampus (red bars) compared to that in the α2^+/+^ mice after treatment with PBS or LPS. **a**, **b** TaqMan quantitative PCR analysis of *Tlr4* relative to the expression of *β-actin* in the hypothalamus (**a**) and hippocampus (**b**) showed no difference between the α_2_^+/+^ and α_2_^+/G301R^ animals. The data are presented as the mean ± SEM (two-way ANOVA followed by Tukey’s post hoc test *n*=3-4).

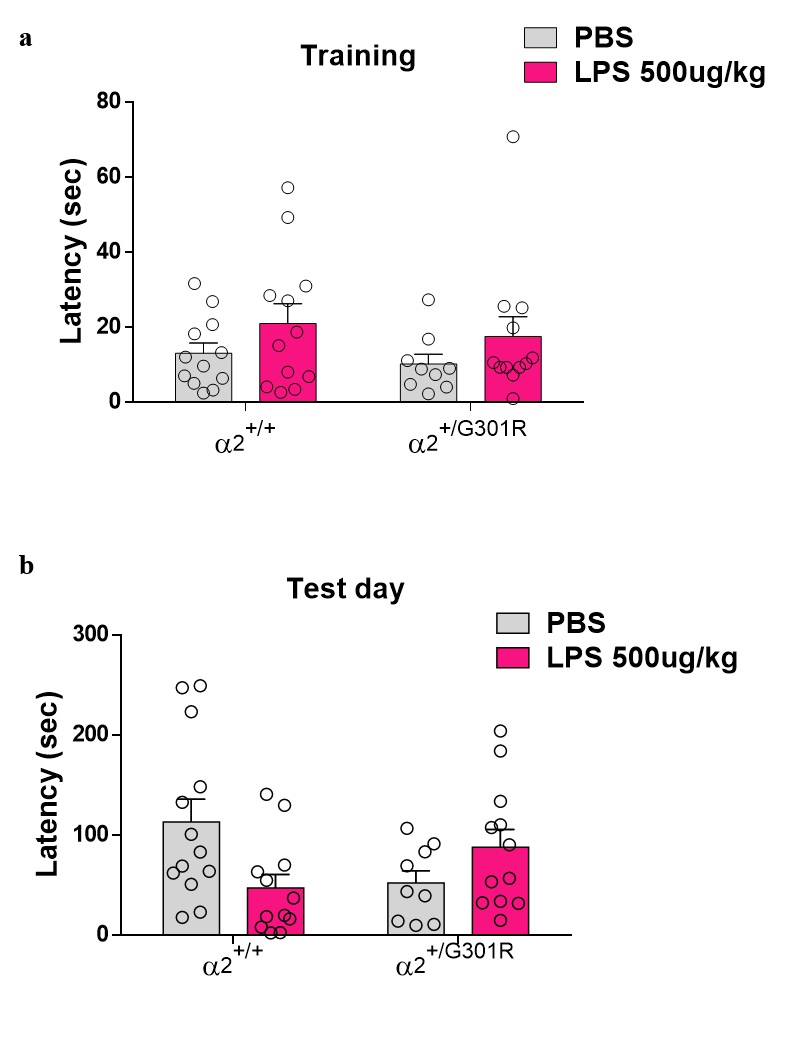


**Supplementary Figure 9.** **a** All groups show no difference at baseline to enter the dark chamber in the training stage (before LPS challenge). **b** There is no difference between all groups to enter to dark chamber after LPS challenge. The data are presented as mean ± SEM (Kruskal-Wallis test followed by Dunn’s post hoc test n=10-12).
